# Treatment of patients with metastatic renal cell carcinoma undergoing hemodialysis: case report of two patients and short literature review

**DOI:** 10.1186/1471-2369-14-84

**Published:** 2013-04-12

**Authors:** John Syrios, Georgios Kechagias, Nicolas Tsavaris

**Affiliations:** 1 Oncology Unit, Department of Pathophysiology, Laikon General Hospital, Athens University School of Medicine, 75 M. Asias str, Athens, 115 27, Greece

**Keywords:** Bilateral renal cell carcinoma, Hemodialysis, Nephrectomy, Targeted agents, Tyrosine kinase inhibitor

## Abstract

**Background:**

Renal cell carcinoma (RCC) may involve both kidneys. When bilateral nephrectomy is necessary renal replacement therapy is mandatory. Treating such patients with sequential therapy based on cytokines, antiangiogenic factors and mammalian target of rapamycin (mTOR) inhibitors is challenging.

**Case presentation:**

The first case, a 50-year-old Caucasian female, underwent a radical right nephrectomy for RCC. Twelve years later she underwent a radical left nephrectomy along with total hysterectomy including bilateral salpingo-oophorectomy for RCC involving the right kidney and ovary. Hemodialysis was necessary because of bilateral nephrectomy. She relapsed with pulmonary metastases and enlarged mediastinal lymph nodes and received cytokine based therapy along with bevacizumab. Therapy was discontinued despite the partial response because of hemorrhagic gastritis. Therapy was switched to an antiangiogenic factor but the patient manifested a parietal brain hematoma and stopped therapy. Subsequently disease relapsed with malignant pleural effusion and pulmonary nodules and a mammalian target of rapamycin inhibitor was administered which was withdrawn only at patient’s deteriorating performance status. The patient died of the disease 13 years after the initial diagnosis of RCC.

The second case, a 51-year-old, Caucasian male, underwent a radical right nephrectomy for a chromophobe RCC. Six months later he underwent a radical left nephrectomy for RCC that proved to be a clear cell RCC. Due to bilateral nephrectomy hemodialysis was obligatory. Following disease recurrence at the anatomical bed of the right kidney therapy with antiangiogenic factor was administered which led to disease regression. However the patient experienced a left temporal-occipital brain hematoma. A radical excision of the recurrence which histologically proved to be a chromophobe RCC was not achieved and the patient received mTOR inhibitor which led to disease complete response. Nine years after the initial diagnosis of RCC he is disease free and leads an active life.

**Conclusion:**

Patients with RCC are in significant risk to manifest bilateral disease. Renal insufficiency requiring hemodialysis poses therapeutic challenges. Clinicians must be aware of the antiangiogenic factors’ adverse effects, especially bleeding, that may manifest in higher frequency and more severe in this setting.

## Background

Renal cell carcinoma (RCC) accounts for 2–3% of all malignant tumors in adults and in Europe represents the third most prevalent urologic malignancy [1]. Metastatic RCC (mRCC) is an aggressive tumor that if left untreated confers a 5 year survival of 0–18% [2]. At the time of diagnosis, one third of the patient presents with locally advanced or metastatic disease and one third of patients undergoing cytoreductive nephrectomy will experience relapse and develop metastasis [3].

The main histological subtypes of RCC are clear cell (75–85% of tumors), papillary, chromophobe, oncocytic and collecting duct carcinomas, which are associated with specific cytogenetic and molecular abnormalities [4]. Clear cell RCC typically carries the 3p deletion and is associated with von Hippel-Lindau disease [5]. Although most RCCs are sporadic, several syndromes associated with RCC have been described. Bilateral RCC’s either synchronous or metachronous are associated with a hereditary predisposition [6,7].

Nephrectomy remains the cornerstone of treatment. It is a prerequisite when the intention is to offer a radical cure to the patient and is usually performed even in the setting of mRCC, except for poor prognosis patients according to MSKCC criteria [8]. In recent years nephron-sparing surgery has largely substituted nephrectomy for small renal tumors and is indicated in case of bilateral tumors whenever feasible [9].

The standard therapy for mRCC beyond cytoreductive surgery is currently based on tyrosine kinase inhibitors (TKI’s) and mammalian target of rapamycin (mTOR) inhibitors which prolong overall survival to 24 months [10].

Patients with mRCC who have severe renal insufficiency at diagnosis and those under hemodialysis following bilateral nephrectomy pertain to a specific group that poses therapeutic challenges to medical oncologists. Since urinary excretion is a major elimination pathway for many antineoplastic drugs, renal impairment may alter the excretion rate of chemotherapeutic agents. Furthermore, in patients undergoing hemodialysis the drug clearance by dialysis must be taken into account for appropriate timing and dosage of chemotherapy. Nonetheless, there are no established guidelines about the management of chemotherapy administration and toxicity in patients undergoing dialysis [11,12]; albeit both TKI’s and mTOR inhibitors have mainly hepatic metabolism and only a minor renal excretion [13-16].

In this case report and short literature review we present 2 patients with bilateral RCC who underwent bilateral nephrectomy and received therapy based on cytokines, antiangiogenic factors, inhibitors of tyrosine kinases receptors and inhibitors of the mammalian target of rapamycin while on hemodialysis.

## Case presentation

The first case, a 50-year-old Caucasian female with a medical history of insulin-dependent diabetes mellitus and hypertension underwent in 1996 a radical left nephrectomy for RCC grade 3, stage T3N0, clear cell carcinoma which was revealed on routine abdominal ultrasound (US) exam. Because of persistent emesis in July 2008 she underwent a gastroscopy and an abdominal computed tomography scan (CT) which was significant for a large mass in the right kidney consistent with renal cell carcinoma and for a second one in her right ovary. Given the large volume of the tumor that invaded the renal vein a nephron-sparing procedure was not feasible. A radical nephrectomy along with total abdominal hysterectomy (TAH) with bilateral salpino-oophorectomy was performed. Pathology assessment of the nephrectomy specimen revealed a Fuhrman grade 3, clear cell renal carcinoma that invaded the Gerota fascia, the renal vein and 1 out of 8 para-aortic lymph nodes, staged as T4N1. The TAH specimen revealed an invaded by the same renal clear cell carcinoma right ovary. Because of a high preoperative serum creatinine and urea the patient required renal replacement therapy which was continued postoperatively 3 times weekly. On February 2009, a chest CT scan performed on a regular follow-up basis revealed multiple pulmonary nodules and enlarged aorta-pulmonary lymph nodes. A cytokine based therapy including Interferon alfa-2b 6 MU administered three times per week subcutaneously, along with Bevacizumab 200 mg intravenously weekly, was started. A partial response of the disease which consisted of disappearance of pulmonary nodules and stability of the mediastinal lymph nodes was observed two months after the initiation of the regimen. Nevertheless, on September 2009 the patient complained of hematemesis and melena. A hemorrhagic gastritis on the grounds of angiodysplasia was diagnosed at gastroscopy which forced the discontinuation of therapy. The hemorrhagic gastritis due to angiodysplasia was attributed to Bevacizumab and also to the administration of heparin during dialysis. Upon her recovery from gastric hemorrhage she resumed therapy based on Sunitinib at 50 mg/day for 4 weeks with a 2-week washout phase. However, 3 weeks after Sunitinib administration the patient experienced left hemiparesis along with expressive aphasia, symptoms caused by a right parietal hematoma as shown on brain CT scan. The brain magnetic resonance imaging (MRI) performed subsequently verified the findings of the CT scan and therapy based on antiangiogenic factors was permanently withheld. Only after the disease relapsed with malignant pleural effusion and pulmonary nodules on December 2009 did the patient resume therapy based on a second generation mTOR inhibitor. The patient was on Everolimus 10 mg/day per os until March 2010 when therapy was discontinued because of the patient’s deteriorating performance status. She died of the renal carcinoma on May 2010, 13 years after the initial diagnosis of RCC.

The second case, a 51-year-old Caucasian male, a heavy smoker, complained of abdominal discomfort and an abdominal ultrasound (US) exam performed in June 2004 was significant for a mass in the right kidney, consistent with RCC. He underwent a radical right nephrectomy that was histologically proved to be a chromophobe RCC of nuclear grade Fuhrman 4, stage T1N0. Following regular follow-up with abdominal US in December 2004, a solid mass in his left kidney was revealed, which was again consistent with RCC. He underwent a radical left nephrectomy and the lesion histologically proved to be a clear cell RCC, of nuclear grade Fuhrman 3, stage T1N0. The patient required renal replacement therapy and he started a three times weekly course of hemodialysis. In December 2009 after a regular basis follow-up with abdominal CT scan, an asymptomatic local disease recurrence at the anatomical bed of the right kidney consisting of a solid mass of 34 mm diameter with central calcification, displacing the inferior vena cava and psoas muscle was diagnosed. Sunitinib at 50 mg/day for 4 weeks with a 2-week washout phase was then administered leading to partial disease response. A radical excision of the recurrence was attempted in January 2010, however the surgical margins were macroscopically invaded by RCC. Interestingly, the lesion was histologically proved to be a recurrence of the primary chromophobe RCC. Two weeks after the resection the patient manifested a subacute left temporal-occipital hematoma in brain CT scan which was attributed mainly to a hemostasis disorder induced by the dialysis procedures. Due to postoperative residual disease the patient was treated with a second generation mTOR inhibitor, Everolimus 10 mg/day per os which led to complete response of the disease, without major toxicity. Nine years after the initial diagnosis of RCC he is disease free and leads an active life.

### Discussion

The incidence of bilateral RCC either synchronous or metachronous has been reported to be 2–13% [17]. Patients with RCC are in high risk to manifest a second RCC either in the affected kidney in case of partial nephrectomy or in the contralateral one [7]. In Rabbani’s et al. study [18] the incidence of metachronous contralateral RCC was stable on long-term follow-up; however it was strikingly high during the first 5 years of follow-up.

Although most RCCs are sporadic, several syndromes associated with bilateral RCC have been described [6,7]. Hereditary renal cancers are usually multiple, bilateral and often occur at a young age. In support of this epidemiologic observation, Klatte et al. [19] reported that among patients with bilateral RCC, familial predisposition was found in 14.3% of them. Additionally, the von Hippel-Lindau (VHL) disease was observed in 4.3% of cases. In terms of pathology, the clear cell RCC typically carries the 3p deletion and is associated with VHL disease [5].

The first patient of the study was diagnosed in 1996 with a clear cell RCC of the left kidney, grade 3, stage T3N0M0. The patient suffered from diabetes mellitus which has not been proved a risk factor for RCC; however, diabetes induced hypertension is an established risk factor for developing RCC [20]. In 2008 she developed a second clear cell RCC at the contralateral kidney. The fact that metachronous RCC appeared 12 years after the primary diagnosis is consistent with the suggestion of Rabbani et al. [18] that surveillance of the contralateral kidney should remain rigorous on extended follow-up. Interestingly, the second RCC patient of the study, a young Caucasian heavy smoker male, recurred in the first 6 months from the diagnosis of the primary lesion fulfilling the temporal criteria for synchronous cancer; however the contralateral lesion was of a different histology. The histological discrepancy between the two synchronous tumors is an extremely rare phenomenon which led us to regard the second lesion as a second primary.

Patients with bilateral or multifocal RCC are in high risk to develop renal insufficiency. Over the past decade an increasing number of authors advocated the nephron sparing surgery to overcome the risk of end stage renal disease and the need for renal replacement therapy [9,21]. Currently, partial nephrectomy is recommended for small lesions <7 cm without compromising overall survival. It is also the treatment of choice in case of bilateral RCC, non-functional contralateral kidney, solitary kidney; however, surgical feasibility is a major criterion for partial nephrectomy [9].

In the cases where nephron sparing technique is not technically feasible, radical open or laparoscopic nephrectomy is required. In such circumstances patients undergo renal replacement therapy, either hemodialysis or peritoneal dialysis; however a possible alternative in disease free patients is kidney transplantation [22].

In both patients of the present study dialysis was mandatory due to bilateral nephrectomy. Treating patients under renal replacement therapy is challenging and scant literature dealing with this issue is currently available. In patients who undergo dialysis the excretion of drugs that normally have a renal excretion follow the dialysis’ clearance rate. It is therefore important to evaluate the fraction of active substance removed by dialysis in order to plan therapy and avoid major toxicity. On the other hand, for drugs that are not excreted by dialysis administration can take place even shortly before the dialysis. There are three indices to estimate the influence of hemodialysis on drug pharmacokinetics, which include the dialysis clearance, the extraction ratio, and the dialysis extraction factor coefficient [23]. Janus’s et al. [11] study addresses the question on dosage adjustment and timing of chemotherapy in hemodialyzed patients; however, no information is provided about the newer agents used in mRCC treatment.

The current medical armamentarium for metastatic RCC includes cytokines (including interferon-α and interleukine-2), antiangiogenic factors that inhibit directly VEGF (Bevacizumab), others that target VEGF receptors and tyrosine kinases receptors (sorafenib, sunitinib, pazopanib, axitinib) and factors that inhibit the mammalian target of rapamycin (temsirolimus and everolimus) [24]. With the development of these agents, the progression-free survival (PFS) has practically doubled and up to 30% of patients achieve partial remission (PR) [25]. In a cohort of 336 mRCC patients treated exclusively with targeted agents the median overall survival (OS) was 24 months (95% CI 20.0, 27.0) and the 5-year OS rate was 24.6% (95% CI 18.7, 30.8%). Interestingly, patients of all prognostic groups participated in this study [10].

Nonetheless, no patients undergoing hemodialysis were included in these studies highlighting the scarcity of data in this clinically relevant minority of patients.

Interferon alfa-2b has a primarily renal metabolism. Evidence based on case reports suggests that RCC patients undergoing dialysis receive low dose Interferon alfa-2b [26] or modify the interval between injections [27]. The first patient of the study received Interferon alfa-2b 6 MU administered subcutaneously three times per week along with Bevacizumab 200 mg intravenously weekly, which was discontinued due to hemorrhagic gastritis partly attributed to Bevacizumab.

Bevacizumab is a monoclonal antibody that inhibits the vascular endothelial growth factor (VEGF). There is only one study [28] evaluating the pharmacokinetics of bevacizumab in a mRCC patient requiring hemodialysis. In this study Bevacizumab was instituted at a dose of 5 mg/kg every 2 weeks and its pharmacokinetic parameters were similar to the reference values of patients with normal renal function. Moreover, bevacizumab is not dialyzable and it may therefore be administered even before dialysis. Further information can be retrieved by studies on patients with metastatic colorectal cancer under dialysis, receiving FOLOFOX/FOLFIRI along with bevacizumab [29,30] with no dose reduction or toxicity from the antibody described. The first patient of the study manifested significant hemorrhagic gastritis while she was on Bevacizumab, so treatment was withheld. Given the fact that in gastroscopy extensive gastric angiodysplasias were found, the gastrointestinal hemorrhage manifesting as hematemesis was co-attributed to Bevacizumab, taking also into account the increased risk for gastric angiodysplasia in end-stage renal disease (ESRD) patients undergoing dialysis.

Sunitinib inhibits the receptor tyrosine kinases (RTKs) VEGF, VEGFR2, PDGFR, FLT-3 and c-KIT and seems to be well tolerated in patients on hemodialysis. In a published case report of 2 patients with ESRD receiving repeated doses of sunitinib (daily for four weeks on, two weeks off) for renal cell cancer, the pharmacokinetics of sunitinib were similar to those of patients with normal renal function [13]. Another report included 10 patients undergoing dialysis with doses starting from 25 to 50 mg daily for 4 out of 6 weeks. Treatment efficacy was comparable to that reported in patients with normal renal function while dose reduction for toxicity was required in 8, but only 1 patient required treatment discontinuation [31]. A phase I study indicated that the dose of sorafenib, an inhibitor of multiple RTKs including VEGF-2, FLT-3, PDGF, FGFR-1 and Raf, should be reduced to 200 mg twice daily for patients on hemodialysis [15]. Masini et al. [32] concur that sunitinib and sorafenib treatment are not contraindicated in patients with mRCC undergoing dialysis since the outcome is similar to that observed in patients with normal renal function. The patients of our study received Sunitinib at 50 mg/day for 4 weeks with a 2-week washout phase but both experienced major bleeding events, such as brain hematomas. Treatment was withdrawn in both cases and the cause of hematomas seemed to be multifactorial. Both patients received therapy the day after dialysis and their serum urea was always above normal values. Moreover, in routine hemodialysis anticoagulation measures are taken consisting of a standard dose of heparin given as a bolus at the start of the dialysis treatment with a mid-treatment dose to maintain suitable anticoagulation. The administration of an antiangiogenetic factor in patients who receive routine anticoagulation during dialysis, could both contribute to a disequilibrium of hemostasis that ultimately could manifest as a multicausal, clinically significant hemorrhage.

Temsirolimus is a parenterally administered inhibitor of the mammalian target of rapamycin (mTOR) kinase. Temsirolimus undergoes hepatic metabolism and is mainly excreted via the feces. Due to the small amount of renal excretion dose adjustment is not needed in the setting hemodialysis [14]. Everolimus is an orally administered mTOR inhibitor. It is extensively metabolized in the liver via CYP3A4 and is excreted by 80% in feces and only by 5% in the urine. Recently, Tiery-Vuillemin et al. [16] evaluated Everolimus pharmacokinetic parameters in two patients suggesting that there is no influence of hemodialysis on Everolimus blood concentrations. However, in the first case of their study a dose reduction from 10 mg/day to 5 mg/day was required due to grade 3 asthenia, while in the second patient no dose escalation from 5 mg/day was achieved due to grade 3 dyslipidemia. The second patient of our study received Everolimus 10 mg/day per os which led to complete response of the disease, without any major toxicity.

Since many new targeted drugs such as pazopanib and axitinib are currently at clinicians’ disposal and others including Etaracizumab, Vorinostat, XL880 and Infliximab are under study, it is advisable that current literature be supplemented with studies addressing administration protocols and toxicity surveillance of the newer agents in RCC patients with end-stage renal disease in order to further optimize our current treatment strategy.

## Conclusion

In patients diagnosed with RCC, especially those with young age at diagnosis, the risk to develop a secondary lesion to the contralateral kidney is relatively high. Nephron sparing surgery is the treatment of choice if technically feasible. Patients undergoing hemodialysis because of bilateral nephrectomy pertain to a group that poses therapeutic challenges to clinicians. Since there are no established guidelines on management of therapy administration and toxicity in mRCC patients undergoing dialysis, therapy should be given with caution and increased vigilance for adverse effects. Medical oncologists must be aware of the higher incidence of bleeding disorders in patients undergoing hemodialysis. More studies on mRCC patients treated with agents targeting molecular pathways under hemodialysis are therefore needed.

## Consent

Written informed consent was obtained from the next of kin of the first patient and from the second patient for publication of this case report. A copy of the written consent is available for review by the Series Editor of this journal.

## Competing interests

The authors declare that they have no competing interests.

## Authors’ contributions

JS and GK assisted in the collection and interpretation of clinical and laboratory data, were involved in bibliographic research and drafted the manuscript. NT was the treating physician, coordinated and supervised drafting the manuscript. All authors read and approved the final manuscript.

## Pre-publication history

The pre-publication history for this paper can be accessed here:

http://www.biomedcentral.com/1471-2369/14/84/prepub
